# Recent Advances in Clinical Nutrition in Stroke Rehabilitation

**DOI:** 10.3390/nu14061130

**Published:** 2022-03-08

**Authors:** Yoshihiro Yoshimura

**Affiliations:** Center for Sarcopenia and Malnutrition Research, Kumamoto Rehabilitation Hospital, 760 Magate, Kikuyo-Town, Kikuchi-County, Kumamoto 869-1106, Japan; hanley.belfus@gmail.com; Tel.: +81-96-232-3111; Fax: +81-96-232-3119

Stroke is a common cause of death and disability worldwide. Malnutrition is prevalent in stroke rehabilitation patients, and has serious negative effects on outcomes. In addition, there is growing interest in new concepts related to malnutrition, such as sarcopenia, frailty, cachexia, chronic inflammation, dysphagia, and oral problems, all of which contribute to a poor prognosis. Therefore, it is necessary to assess nutritional status early and, if needed, provide appropriate nutritional intervention to improve patient outcomes. A multidisciplinary approach is strongly recommended in this setting; as such, high-quality clinical evidence regarding clinical nutrition in stroke rehabilitation is needed.

This Special Issue updates our knowledge of clinical nutrition for stroke patients and includes interesting studies on topics including nutrition and weight management in the early stages of stroke, the relationship between frailty and improved physical function, weight gain by providing stored energy, physical activity and diet quality, L-carnitine and cognitive level, and the prediction of stroke prognosis using temporal muscles.

Aggressive nutritional management at the early stages of stroke onset may be effective in improving prognosis. In a retrospective cohort study, Sato et al. showed that high-energy nutritional intake during the first week post-stroke was associated with high rates of discharge from the hospital to home [1]. Additionally, Kishimoto et al. showed that weight maintenance or gain in post-stroke patients during the early phases of convalescence rehabilitation is independently associated with improvements in physical function [2]. Furthermore, Yoshimura et al. conducted a retrospective cohort study of underweight patients aged ≥70 years with a body mass index of less than 20.0 kg/m^2^ undergoing convalescent rehabilitation after stroke. The study found that providing stored energy contributed to weight gain and increased skeletal muscle mass [3], and that it took approximately 9600 kcal of energy to gain 1 kg of body weight in underweight patients. These findings emphasize the importance of not only exercise therapy [4] and correction of polypharmacy [5], but also of aggressive nutritional support at the early stages to improve prognosis post-stroke.

Physical activity is also important in the rehabilitation of stroke patients. Nguyen et al. showed that physical activity and diet quality significantly modified the negative impacts of comorbidity on disability in stroke patients [6]. Comorbidities in stroke patients are strongly associated with poor prognosis, death, increased levels of disability, and worse functional outcomes post-stroke. Therefore, it is important to assess comorbidities early and increase physical activity and diet quality for appropriate treatment and rehabilitation. Furthermore, Nozoe et al. showed that pre-stroke frailty was associated with declines in physical function several months post-stroke [7]. These findings indicate that preventing frailty reduces functional disability, and that maintaining high physical function is associated with a better post-stroke quality of life.

Another interesting finding is that L-carnitine may serve a neuroprotective role against white matter microstructural damage and cognitive impairment in hemodialysis patients, according to Ueno et al. [8]. Long-term administration of carnitine may ameliorate damage to white matter microstructure by suppressing neuroinflammation and improving the executive function and attention associated with the protection of several candidate fiber tracts. Long-term administration of carnitine may be a novel treatment for vascular dementia. However, as this study is based on hemodialysis patients, further studies are needed to validate the neuroprotective effects of L-carnitine in post-stroke patients.

Katsuki et al. outlined reports on temporal muscle thickness (TMT) and stroke. TMT is associated with nutritional status and risk of sarcopenia after stroke. It is also a useful prognostic marker for dysphagia in patients with subarachnoid and cerebral hemorrhage. In recent years, there has been a rapid increase in the number of reports on TMT and stroke, as TMT is considered one of the most important clinical factors [9]. Sarcopenia and malnutrition are frequently observed in stroke patients and are associated with impaired rehabilitation outcomes. Instruments such as bioelectrical impedance analysis and dual energy X-ray absorptiometry are required to evaluate skeletal muscle mass in diagnosing sarcopenia. However, if TMT can be quantified simply by echo, it may be widely applied in daily stroke rehabilitation clinical practice.

The current Special Issue presents recent advances in clinical nutrition in stroke rehabilitation, highlighting the importance of nutritional management and physical activity in improving functional outcomes after stroke. The advances shown are of great interest from a clinical perspective, with a growing number of stroke patients around the world, and may act as the basis for future research.

## Data Availability

Not applicable.
